# Pericardial fat volume and coronary atherosclerotic markers among body mass index groups

**DOI:** 10.1002/clc.23396

**Published:** 2020-06-03

**Authors:** Mohammed Bader Hassan, Hussein Nafakhi, Abdulameer A. Al‐Mosawi

**Affiliations:** ^1^ Radiology Department Medicine College, Aliraqia University Baghdad Iraq; ^2^ Internal Medicine Department Medicine College, University of Kufa Najaf Iraq; ^3^ Radiology Department Medicine College, University Of Kufa Najaf Iraq

**Keywords:** BMI, coronary atherosclerosis, CT angiography, obesity, pericardial fat

## Abstract

**Background:**

Increased pericardial fat volume (PFV) is associated with coronary atherosclerosis burden independent of body mass index (BMI) in many clinical studies. However, the association of PFV with markers of coronary atherosclerosis has not yet been assessed by dividing the patients according to BMI categories.

**Hypothesis:**

To assess the association of PFV measured by multi‐detector CT (MDCT) angiography with coronary atherosclerotic markers (coronary artery calcium score [CAC], plaque type, and luminal stenosis) among BMI categories.

**Methods:**

A total of 496 patients with suspected coronary artery disease who underwent 64‐slice MDCT angiography examination were enrolled. Patients divided into obese, overweight, and normal weight groups according to BMI degree.

**Results:**

PFV showed a significant association with CAC, non‐calcified coronary plaque, and significant coronary stenosis in obese group. After adjusting for cardiac risk factors, the association of PFV with the non‐calcified coronary plaque and significant coronary stenosis persisted. There was a significant association between PFV with CAC and significant coronary stenosis in normal weight group. The association between PFV with CAC and significant coronary stenosis in normal weight was persisted afar adjusting for cardiac risk factors. No significant association was noted between PFV with coronary plaque type in normal weight group. There was no significant independent association between PFV with coronary atherosclerotic markers in overweight group.

**Conclusions:**

Increased PFV was associated with advanced stage atherosclerosis in normal weight group, while increased PFV was associated with non‐calcified plaque in obese. These results highlight the differential relationship of PFV with coronary atherosclerotic markers among BMI categories.

## 1 INTRODUCTION

Obesity is a heterogeneous disease associated with the clustering of several cardiac risk factors and different degrees of cardiovascular and metabolic disturbances.^1^, ^2^

In the literature, obesity‐related cardiometabolic disturbances have been associated with coronary atherosclerosis and the development of poor long‐term prognosis.^1^

However, the overall link between obesity measured by body mass index (BMI) and coronary atherosclerosis is complex and rather weak in recent cohort studies. BMI does not take into account the anatomical variance, composition, and function of body adipose tissues or distinguish lean body mass from the actual adipose mass.^3^

In the last two decades, pericardial fat deposition has been reported to have a major physiological and metabolic role in the pathogenesis of coronary atherosclerosis owing to its local proximity to coronary vasculatures and the systemic influence of bioactive substances secreted by the pericardial fat cells. Many clinical studies have reported the pathophysiological link between increased pericardial fat volume (PFV) and coronary atherosclerosis independent of BMI and beyond general obesity.^4^, ^5^, ^6^

Recently, several clinical studies reported inconsistent results regarding the association of increased pericardial fat deposition with coronary atherosclerotic markers. Such inconsistent or controversial relationships might be due to lack of dividing the patients according to BMI degree, as the pathophysiological interaction between increased PFV and coronary atherosclerosis burden may be associated with various metabolic and circulatory factors of obesity.^3^, ^7^, ^8^

To address the potential confounding effect of BMI grades on the association of PFV with coronary atherosclerosis burden, we aimed to assess the possible relationships of PFV measured by multi‐detector CT (MDCT) angiography with coronary atherosclerotic markers (coronary artery calcium score [CAC], plaque, and luminal stenosis) among BMI categories.

## 2 METHODS

A cross‐sectional retrospective study was carried out on 496 patients who were referred for 64‐slice MDCT angiography examination to exclude occlusive coronary artery disease at Cardiology Center at Al‐Sader Teaching Hospital between January 2013 and December 2018.

Data related to the individual's cardiac risk factors were obtained at the time of MDCT examination as in our prior study.^6^

BMI calculated as weight (kg)/height (m)^2^ and patients divided into obese (BMI of ≥30 kg/m^2^), overweight (BMI of 25‐29.9 kg/m^2^) and normal weight (BMI of <25 kg/m^2^) groups.

Verbal informed consent obtained from recruited patients. Approval of this study was provided by our medicine college board.

### 2.1 MDCT procedure and quantitative analysis

**FIGURE 1:**
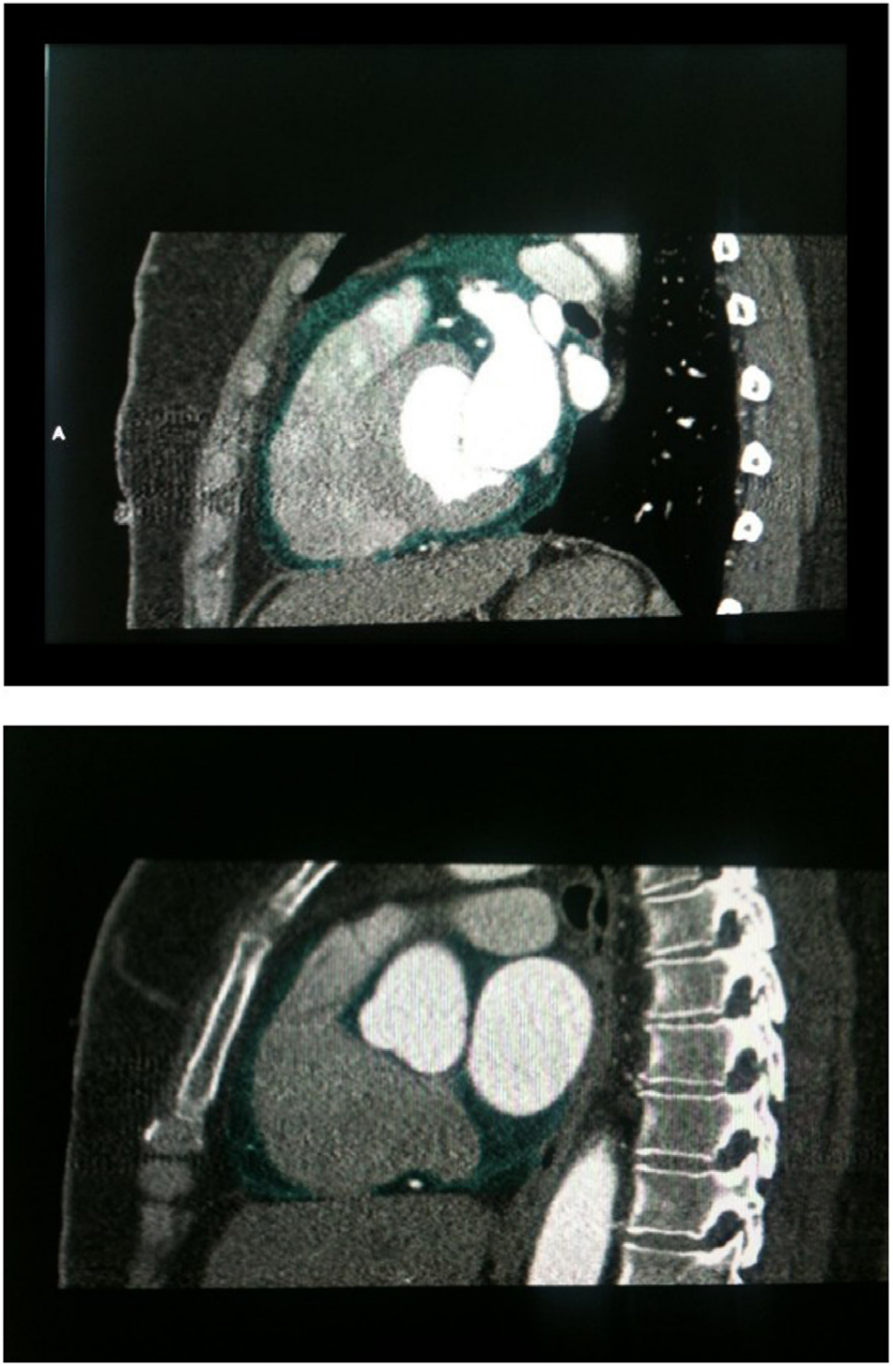
Assessment of PFV by MDCT (green color) at sagittal section of the heart

**FIGURE 2:**
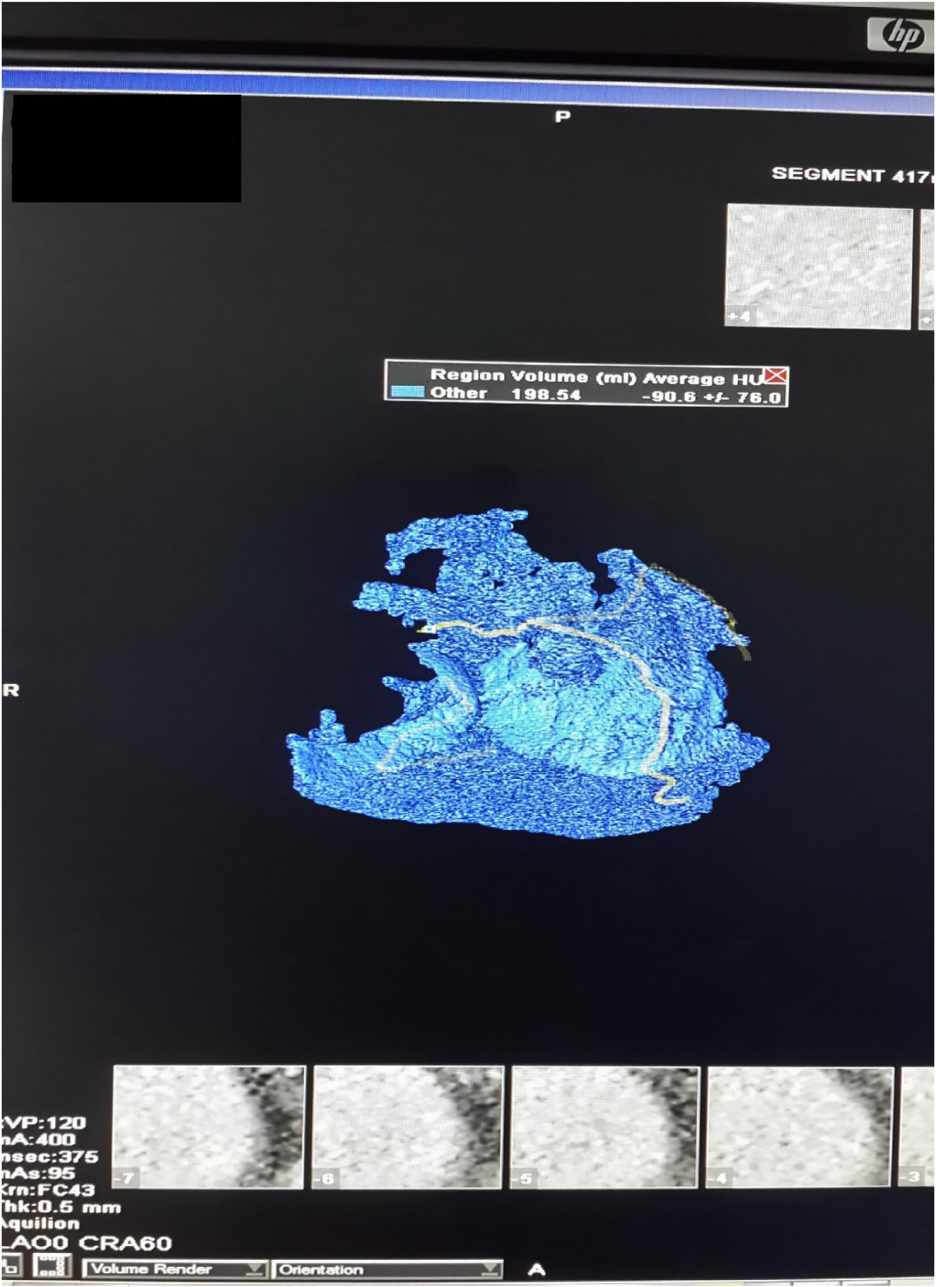
Volume rending measurement of PFV

The used 64‐slice MDCT coronary angiography was described in detail elsewhere.^6^ Pericardial fat volume, measured in a cubic centimeter (cm^3^), was corresponding to fat tissue within the pericardial sac excluding fat outside the parietal pericardium (paracardial fat), as in Figures 1 and 2. PFV and CAC assessed as per our prior study.^6^

The coronary plaque was considered present when there was thickening ≥1 mm in thickness within or adjacent to the coronary artery wall. Plaques classified into calcified (plaque consisting of only calcium or containing both calcified and non‐calcified components) and non‐calcified plaque (plaque that was free of calcium).^6^

Stenosis severity expressed as normal to non‐significant (with a mean lumen diameter reduction of <50%) and significant with a mean lumen diameter reduction of ≥50%.^6^

The MDCT images were analyzed by two radiologists, who were blind to the patient's identity, in consensus. Both of them have more than 5 years of experience in MDCT angiography images analysis.

### 2.2 Statistical analysis

Statistical analysis was performed using SPSS ver. 13.0 (SPSS Inc., Chicago, Illinois). Clinical and MDCT data were expressed as mean ± SD or as numbers with percentages for normally distributed data. Nonnormally distributed data, including PFV and CAC, expressed as median (inter‐quartile range [IQR]). Spearman's rank correlation for nonparametric data was used to assess the correlation between PFV with CAC. The association of PFV with coronary plaque type and coronary stenosis severity was assessed using nonparametric test (Mann‐Whitney *U* test). Multivariate regression analysis was performed to evaluate the association of PFV with CAC and binary logistic regression for the association of PFV with significant coronary stenosis and non‐calcified coronary plaque after adjusting for cardiac risk factors which achieved statistical significance in univariate analysis. A *P*‐value of <.05 was chosen for statistical significance.

## 3 RESULTS

Four hundred and ninety‐six patients (age: 54.6 ± 10 years, 51% males) who underwent MDCT examination to exclude occlusive coronary disease were recruited in the study. The median (IQR) of PFV was 93 (61‐140) cm^3^. According to the BMI category, 93 patients were normal weight (19%), 198 were overweight (40%), and 205 were obese (41%).

### 3.1 Obese group

The mean age was 55 ± 9 years, with 38% males. PFV median (IQR) was 115 (79‐155) cm^3^. The distribution of a family history of premature coronary artery disease, hypertension, and female sex was more prevalent in the obese group compared to the normal weight and overweight groups. Obese patients tended to have increased PFV values compared to normal weight and overweight patients, as in Table 1.

**TABLE 1:** Patient's characteristics

Variables	Normal weight n = 93	Overweight n = 198	Obese n = 205	*P*
Age (years)	53 ± 11	55 ± 11	55 ± 9	.575
Male	59 (63%)	115 (58%)	78 (38%)	<.001
Hypertension	37 (42%)	86 (43%)	138 (70%)	<.001
Diabetes mellitus	10 (11%)	50 (25%)	42 (22%)	.012
Smoking	15 (17%)	47 (24%)	42 (22%)	.382
Family history	13 (15%)	40 (20%)	56 (30%)	.021
Dyslipidemia	10 (11%)	44 (22%)	40 (21%)	.067
CAC	5 (0‐52)	0 (0‐67)	1 (0‐87)	.251
PFV	80 (50–121)	84 (58–127)	115 (79–155)	<.001
Coronary plaques				
Absent	46 (49%)	116 (59%)	104 (50%)	.133
Calcified	45 (49%)	71 (36%)	86 (42%)	.032
Non‐calcified	2 (2%)	11 (5%)	15 (8%)	.263
Coronary stenosis grades				
Non‐significant	26 (28%)	39 (20%)	35 (17%)	.033
Significant	29 (31%)	62 (31%)	78 (38%)	.242

Abbreviations: CAC, coronary artery calcium; PFV, pericardial fat volume.

Increased PFV showed a significant association with CAC (*r* = .212, *P* = .001), non‐calcified coronary plaque (*P* = .001) and significant coronary stenosis (*P* < .001) as in Table 2 and Figures S1 and S2. After adjusting for cardiac risk factors that achieved statistical significance in univariate analysis, the significant association of increased PFV with the non‐calcified coronary plaque and significant coronary stenosis persisted, while PFV association with CAC not persisted, as in Table 3.

**TABLE 2:** Correlations of PFV with coronary stenosis severity among BMI groups

	PFV	
	Median (IQR)	*P*
Normal weight		
Non‐significant stenosis	69 (41‐94)	<.001
Significant stenosis	130 (84‐182)	
Overweight		
Non‐significant stenosis	76 (56‐124)	.032
Significant stenosis	97 (70‐135)	
Obese		
Non‐significant stenosis	95 (60‐146)	<.001
Significant stenosis	132 (114‐178)	

Abbreviations: CAC, coronary artery calcium; IQR, inter‐quartile range; PFV, pericardial fat volume.

**TABLE 3:** Regression analysis

Coronary atherosclerotic markers	Normal weight		Overweight		Obese	
	PFV		PFV		PFV	
	*β* (CI)	*P*	*β* (CI)	*P*	*β* (CI)	*P*
CAC	0.8 (1.0‐1.3)	.012	1 (0.9‐1.0)	.672	1 (0.9‐1.0)	.074
Non‐calcified plaque	1 (0.9‐1.0)	.123	1 (0.9‐1.0)	.549	1 (1.0‐1.1)	.003
Significant coronary stenosis	1 (1.0‐1.1)	.001	1 (0.9‐1.1)	.652	1 (1.0‐1.1)	.004

Abbreviations: CAC, coronary artery calcium; CI, 95% confidence interval; PFV, pericardial fat volume.

### 3.2 Overweight

The mean age was 55 ± 11 years with 58% males. PFV median (IQR) was 84 (58‐127) cm^3^. Diabetes mellitus was more prevalent in the overweight group (*P* = .012) compared to obese and normal weight groups, as in Table 1. Increased PFV showed a significant association with CAC (*r* = .225, *P* = .003) and significant coronary stenosis (*P* = .032), while no significant association was observed between increased PFV with coronary plaque type (calcified or non‐calcified) (*P* = .180), as in Table 2 and Figures S3 and S4. After adjusting for cardiac risk factors, the association of increased PFV with CAC and significant coronary stenosis presence not persisted, as in Table 3.

### 3.3 Normal weight

The mean age was 53 ± 11 years, with 63% males. PFV median (IQR) was 80 (50‐121) cm^3^. Male sex was more prevalent in the normal weight group in comparison to obese and overweight groups (*P* < .001), as in Table 1. Increased PFV showed a significant association with CAC (*r* = .344, *P* = .002), as in Figure S5.There was a significant association between increased PFV and significant coronary stenosis (*P* < .001) as in Table 2. The associations between increased PFV with CAC and significant coronary stenosis persisted afar adjusting for cardiac risk factors as in Table 3. There was no significant association between increased PFV with coronary plaque type (calcified or non‐calcified) (*P* = .143), as in Figure S6.

## 4 DISCUSSION

The significant independent association of PFV with CAC and significant coronary stenosis in normal weight patients and the significant independent association of PFV with the non‐calcified plaque presence and significant coronary stenosis in obese patients were the main results of the present study.

A bulk of available clinical evidence supporting the significant direct and indirect role of obesity to cardiovascular disorders, including coronary atherosclerosis.^9^

BMI is the most widely used simple and reproducible tool to define obesity and measure generalized body adiposity in the large epidemiological and observational studies. However, BMI has been criticized for its apparent defect to distinguish efficiently between fat and non‐fat distribution, particularly in the elderly because of aging‐related body fat redistribution.^3^, ^9^, ^10^

Recently, PFV measured by MDCT, as a marker of cardiac adiposity, has been reported to be an accepted tool to assess overall cardio‐metabolic disturbances associated with increased adiposity better than BMI.^9^, ^10^

Emerging evidence supports the notion that PFV is a more useful marker of cardiometabolic derangements associated with different obesity phenotypes than BMI per se, whereby metabolically healthy obesity differs from the metabolically unhealthy obesity in terms of cardiac fat distribution and cardiac risk factors while the metabolically obese but normal weight phenotype shows high visceral fatty tissue percentage and increased cardiometabolic risk, despite the normal BMI.^2^ Thus, the significant contribution of PFV to obesity‐related cardiometabolic risk may explain the risk differential observed in metabolically healthy obese and metabolically obese but normal weight phenotype.^10^

Despite the significant association of coronary atherosclerotic markers with PFV in normal weight patients in the present study, there was a significant difference in the distribution of PFV between obese and normal weight, whereby obese patients tended to have a significant increase in PFV compared to normal weight patients.

It has reported that obese patients showed a higher propensity than normal weight patients for visceral fat deposition, including pericardial fat, that is associated with cardiac dysfunction.^3^, ^11^

On the other hand, several studies showed that PFV is associated with cardiac changes in normal weight patients with subclinical coronary atherosclerosis.^12^, ^13^

Fat in the pericardial space tissue has been suggested to have complex dichotomous functional characteristics, adverse, and protective, interacting with the coronary vessels. However, it is not clear whether pericardial fat changes precede or follow coronary atherosclerosis development or how pericardial fat expansion is regulated.^11^

Besides that, there is a piece of clinical evidence supporting the presence of fat tissue anatomical and functional disturbances regardless of increased fat tissue amount or increased general weight. Hence, the presence of diseased fat tissue regardless the presence of increased fat deposition can contribute to increased cardiometabolic risk.^14^

In the present study, the significant association of PFV with non‐calcified plaques, rather than calcified plaques, in obese has raised an important inquiry whether this relationship may be associated with increased risk of acute coronary events resulting from the erosion or rupture of these high risk and vulnerable plaques.^15^

In general, pericardial fat accumulation may precede plaque calcification and the development of mature atherosclerotic plaques.^16^

Recently, Bamberg et al reported that non‐calcified plaques measured by MDCT are a feature of early‐stage atherosclerosis and that their presence decreases with increasing age.^17^

Along the same line, Isma'eel et al showed that obesity was 2.76 times more likely to be associated with a non‐calcified plaque in patients with zero CAC as compared to normal weight.^18^

In supporting the above results, Imai et al found that progression of non‐calcified plaque correlated with increased visceral fatty tissue deposition and higher BMI quartiles, independent of conventional cardiac risk factors.^19^

Taken together, the findings of the present study and data from the studies, as mentioned above, may suggest that increased PFV may be present even with no increased CAC and before plaque calcification occurs. Also, pericardial fat deposition may precede the development of more advanced atherosclerosis. Hence, the association of increased PFV with a non‐calcified plaque in obese patients may be associated with an increased risk of acute coronary events in the future.^16^

In agreement with Yong et al^20^ results, PFV showed no statistically significant independent association with coronary atherosclerotic markers among overweight patients in the present study.^20^ Also, some studies reported that overweight patients had significantly lower cardiovascular and all‐cause mortality relative to normal weight and obese patients.^21^, ^22^ ^23^ possible explanations for the statistically non‐significant association between PFV with coronary atherosclerotic markers in the present study and lower cardiovascular mortality noted among overweight patients in other studies have included earlier presentation of overweight and mildly obese patients, greater likelihood of receiving investigations and interventions at earlier stage in the disease process, selection bias as the study population was based on patients who underwent MDCT examination and differential cardio‐protective metabolic reserve of increased body fat in overweight and mildly obese persons to offset the adverse effects of obesity.^22,23^

Some limitations need to be taken into account in the present study. First, it was a single‐center study with a possibility of selection bias, and this study did not include long‐term follow‐up. Second, we did not assess inflammatory markers or adipokines, which could have an important role in the pathogenesis of early‐stage coronary atherosclerosis and assessment of the functional status of pericardial fat. Third, additional measures of obesity‐related cardiometabolic burden, such as muscle mass or waist circumference were not performed in the present study. Fourth, some high‐risk features related to coronary plaque characteristics, such as positive remodeling or plaque vulnerability, were not investigated due to the retrospective design of the present study. Follow‐ups studies are required to determine the prognostic role of increased PFV and early detection of subclinical coronary atherosclerosis.

## 5 CONCLUSIONS

Increased PFV was associated with calcified and advanced stages of coronary atherosclerosis in normal weight patients compared to obese. In obese, increased PFV associated with non‐calcified plaque presence and this may contribute to an unfavorable coronary risk profile with future risk of acute coronary events as a consequence of plaque erosion. These results highlight the differential relationship of increased PFV with coronary atherosclerotic markers among different BMI grades.

## CONFLICT OF INTEREST

The authors declare that they have no conflict of interest.

## Supporting information


**Figure S1** Association of PFV and CAC among obese patients

**Figure S2** Association of PFV and CAC among overweight patients

**Figure S3** Association of PFV and CAC among normal weight patients

**Figure S4** Relationship of PFV with coronary plaque type among obese patients

**Figure S5** Relationship of PFV with coronary plaque type among overweight patients

**Figure S6** Relationship of PFV with coronary plaque type among normal weight patients
