# Supplementary figures and images for: Pericardial fat volume and coronary atherosclerotic markers among body mass index groups
Source: Clin Cardiol. 2020 Jun 3;43(9):993–8. doi: 10.1002/clc.23396 (PMC7462198; doi:10.1002/clc.23396)

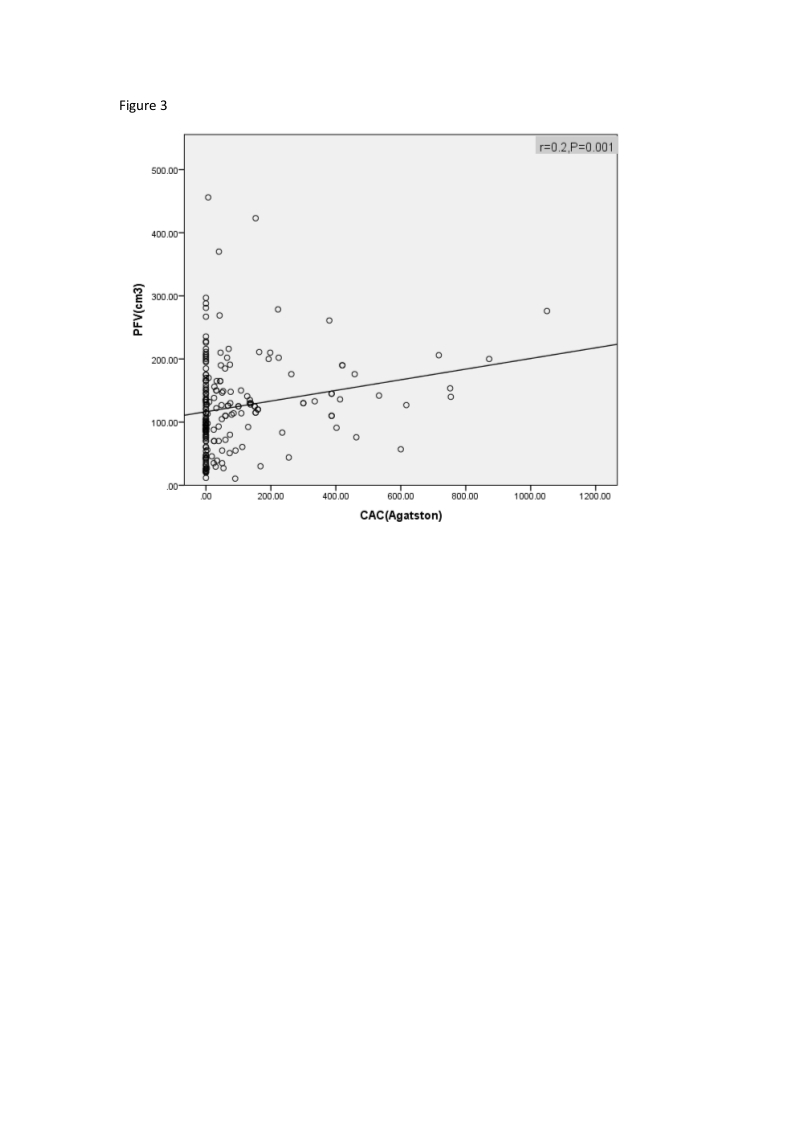

Supplement: Supplementary file 1 — Figure S1 Association of PFV and CAC among obese patients [file CLC-43-993-s001.jpg]

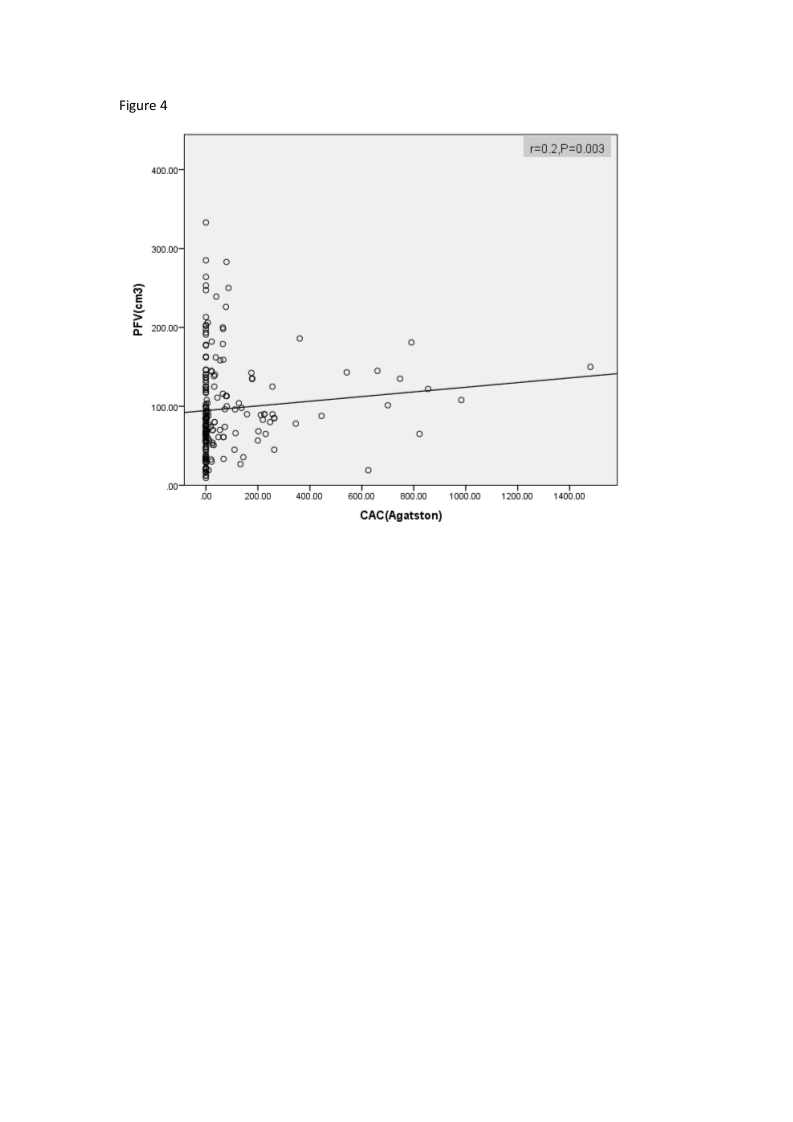

Supplement: Supplementary file 2 — Figure S2 Association of PFV and CAC among overweight patients [file CLC-43-993-s002.jpg]

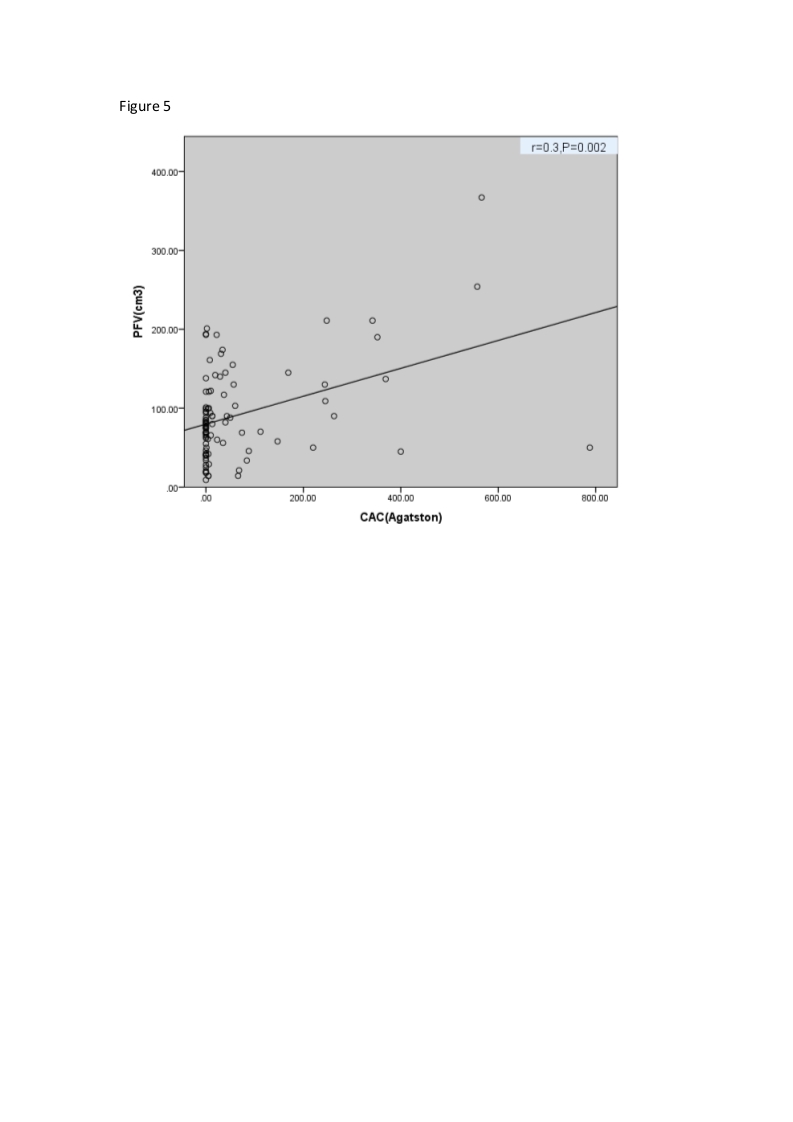

Supplement: Supplementary file 3 — Figure S3 Association of PFV and CAC among normal weight patients [file CLC-43-993-s003.jpg]

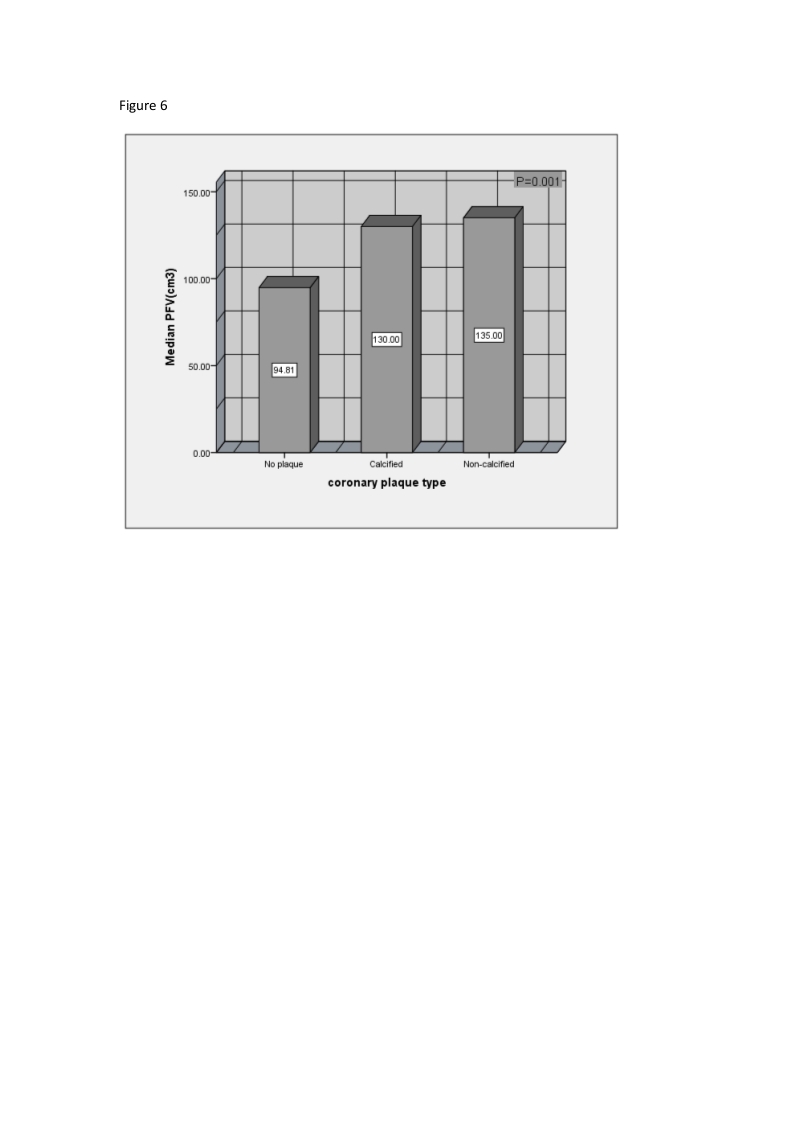

Supplement: Supplementary file 4 — Figure S4 Relationship of PFV with coronary plaque type among obese patients [file CLC-43-993-s004.jpg]

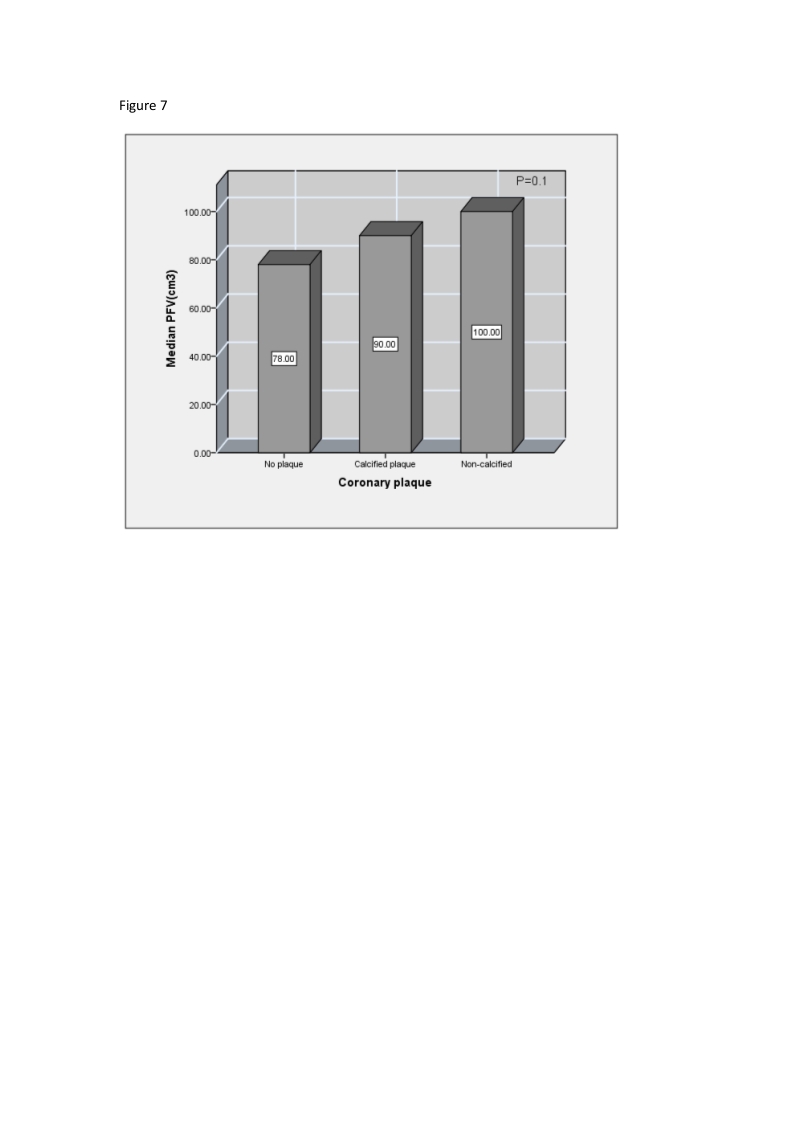

Supplement: Supplementary file 5 — Figure S5 Relationship of PFV with coronary plaque type among overweight patients [file CLC-43-993-s005.jpg]

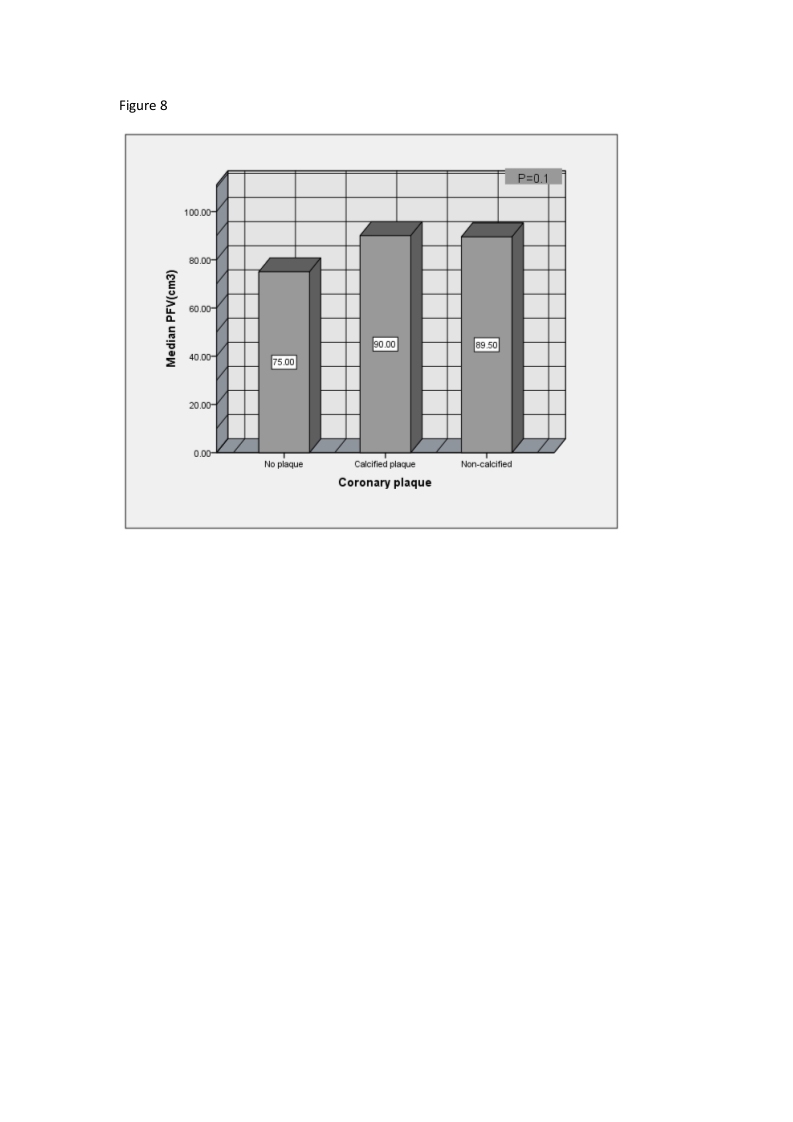

Supplement: Supplementary file 6 — Figure S6 Relationship of PFV with coronary plaque type among normal weight patients [file CLC-43-993-s006.jpg]
